# A trade-off between dry season survival longevity and wet season high net reproduction can explain the persistence of *Anopheles* mosquitoes

**DOI:** 10.1186/s13071-018-3158-0

**Published:** 2018-11-03

**Authors:** Gesham Magombedze, Neil M. Ferguson, Azra C. Ghani

**Affiliations:** 1 Center for Infectious Diseases Research and Experimental Therapeutics, Baylor Research Institute, Baylor University Medical Center, Dallas, TX USA; 2 MRC Centre for Global Infectious Disease Analysis, Department of Infectious Disease Epidemiology, Imperial College London, London, UK

**Keywords:** *Plasmodium falciparum*, *Anopheles* mosquitoes, Vector ecology, Mathematical modelling, Aestivation, Persistence

## Abstract

**Background:**

*Plasmodium falciparum* malaria remains a leading cause of death in tropical regions of the world. Despite efforts to reduce transmission, rebounds associated with the persistence of malaria vectors have remained a major impediment to local elimination. One area that remains poorly understood is how *Anopheles* populations survive long dry seasons to re-emerge following the onset of the rains.

**Methods:**

We developed a suite of mathematical models to explore the impact of different dry-season mosquito survival strategies on the dynamics of vector populations. We fitted these models to an *Anopheles* population data set from Mali to estimate the model parameters and evaluate whether incorporating aestivation improved the fit of the model to the observed seasonal dynamics. We used the fitted models to explore the impact of intervention strategies that target aestivating mosquitoes in addition to targeting active mosquitoes and larvae.

**Results:**

Including aestivation in the model significantly improved our ability to reproduce the observed seasonal dynamics of vector populations as judged by the deviance information criterion (DIC). Furthermore, such a model resulted in more biologically plausible active mosquito survival times (for *A. coluzzii* median wet season survival time of 10.9 days, 95% credible interval (CrI): 10.0–14.5 days in a model with aestivation *versus* 38.1 days, 95% CrI: 35.8–42.5 days in a model without aestivation; similar patterns were observed for *A. arabiensis*). Aestivation also generated enhanced persistence of the vector population over a wider range of both survival times and fecundity levels. Adding vector control interventions that target the aestivating mosquito population is shown to have the potential to enhance the impact of existing vector control.

**Conclusions:**

Dry season survival attributes appear to drive vector population persistence and therefore have implications for vector control. Further research is therefore needed to better understand these mechanisms and to evaluate the additional benefit of vector control strategies that specifically target dormant mosquitoes.

**Electronic supplementary material:**

The online version of this article (10.1186/s13071-018-3158-0) contains supplementary material, which is available to authorized users.

## Background

Despite decades of intensive research and the availability of several disease and vector control intervention tools, malaria, caused by *Plasmodium* spp., remains a leading global public health concern. In 2016 there were an estimated 216 million new cases of malaria worldwide and just under half a million malaria-associated deaths [1]. Ninety percent of malaria deaths are estimated to occur in Africa, the majority in children under the age of five [2]. *Plasmodium falciparum* is the main cause of malaria death in this region, with *Anopheles gambiae* (*s.s.*), *Anopheles coluzzii*, *Anopheles arabiensis* and *Anopheles funestus* being the principal vectors for transmission. These vectors are widespread in the sub-Saharan region and are well adapted to the dry Savannahs and the semi-arid environments where surface water essential for their reproduction is absent for about four to seven months [3–5]. In many parts of Africa, and particularly in the Sahel region, the incidence of disease is therefore highly seasonal and concomitant with vector population density, which fluctuates with rainfall [6, 7]. However, the mechanisms ensuring their survival through the dry season remain elusive [3–5, 8].

Understanding the dry season ecology of the *Anopheles* vectors has been a puzzle for many decades [9–12]. During the seven month long dry season in the Sahel region, temporal water reservoirs dry up, breeding sites vanish and mosquito populations drop to their lowest levels before the rainy season returns [3–5, 8, 13–15]. Irrespective of these harsh conditions, the mosquitoes somehow survive and the return of the rains is accompanied by a surge in their population growth, commensurate with the start of the malaria transmission season. Studies suggest that mosquitoes have the ability to sense the changing environmental conditions and hence make necessary adjustments to evade extinction. There is unequivocal evidence (reviewed in [16]) that demonstrate that several species of insects employ different strategies to survive harsh environmental conditions in their habitats [8, 16–18]. They use several cues, which include sensing the change in photoperiod, temperature (winter/summer), rainfall, availability of food and population density [15–17, 19–23]. These orchestrate a sequence of events that trigger physiological changes that will enable the insects to adapt and therefore enter into a survival mode (a dormant state or hibernation) or to display multiple discrete reversible phenotypes (phenotypic plasticity or polyphenism). This involves a build-up of nutritional reserves ahead of time, reduced metabolism, little or no feeding, cessation of reproduction, modified flying behaviour, halted growth or development, increased desiccation and cold or heat tolerance [8, 14, 15, 17, 18, 24, 25].

Given the repertoire of strategies insects can use and the different life history traits of each biological species/insect, insects can enter diapause at different stages of their life-cycle. It has been demonstrated in studies reviewed in [16, 19, 20] that *Aedes albopictus* can diapause in winter (overwintering) as eggs, *Culex* spp. at several stages of their aquatic life-cycle [21, 22, 26–28], while *Anopheles* mosquitoes diapause in summer (aestivation) as adult vectors [3–5]. It has been suggested that aestivation of *Anopheles* mosquitoes is not triggered by lack of food or by adverse temperatures (which remain high in the Sahel region), but rather by the disappearance of breeding sites [15, 24]. Given that an adult *Anopheles* mosquito would not normally survive longer than four weeks during the wet season and that their aquatic life stages cannot survive without water, aestivation appears a plausible mechanism for persistence.

Migration is another strategy for persistence [29–32], but is probably used by insects that lack the machinery for environmental adaptation and those that do not exhibit phenotypic plasticity traits. Studies suggest that *A. coluzzii* survives the long dry season through aestivation while *A. gambiae* (*s.s.*) and *A. arabiensis* survive by migrating to long distance refugia (where water persists) [3, 5]. Hibernation of female mosquitoes away from human compounds necessitates the modification of their feeding habits if they are to survive. Studies report a switch from feeding on human blood to other food sources such as flower nectar and woody-plant juices [21, 33, 34]. The lack of high protein food meals in part explains the biology of gonotrophic dissociation and concordance that is observed during the dry season in several African malaria vectors; reproduction arrest (reproductive diapause) of the mosquitoes is considered the ultimate hallmark of aestivation [14, 15, 18]. Some studies suggest that gravid mosquitoes potentially wait for the arrival of the rainy season to lay their eggs or can resorb their eggs due to oviposition deprivation [16, 19, 24]. However, because of their plastic traits, the mosquitoes can resume rapid reproduction at the onset of a new rainy season.

Current policies and tools used in vector control are shaped by our knowledge of the relationship between vector emergence and malaria transmission. In particular, prevailing vector control policies recommend the administration of insecticides at the beginning of a rainy season. Intervening at this point will reduce proliferation of the vectors, reduce transmission and therefore reduce the number of new malaria cases. However, an important question is whether this is the best strategy to achieve elimination? Whilst studies have demonstrated that vector control can temporarily achieve vector suppression, in many cases this was short-lived [35–38]. Thus an understanding of dry season persistence is critical to inform vector control strategies for malaria elimination.

Mathematical models have been used extensively to understand mosquito population dynamics [39–41] and evaluate vector control interventions [39, 42, 43]. Here, we use mathematical modelling as a tool to better understand the dry season ecology of the *Anopheles* vectors. We use the models to understand the strategies the mosquitoes employ at the different stages of their life-cycle to survive the dry season and to ensure effective reproduction during the wet season, to understand how these strategies contribute or enable mosquito population persistence, and to explore the consequences of these different strategies for the success of current vector control strategies.

## Methods

### Mathematical model of the mosquito life-cycle

**Fig. 1:**
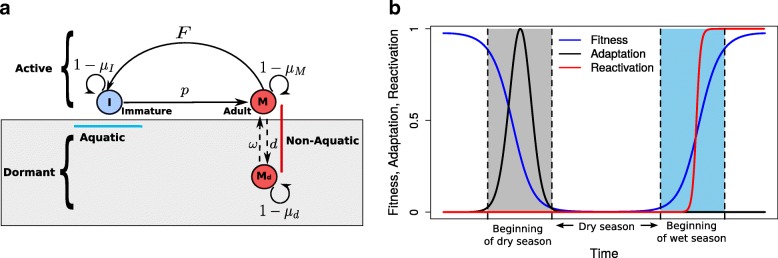
Schematic of the model structure. **a** The modelled life stages. The aquatic stages (eggs, larvae and pupae) are grouped together as immature (*I*) vectors and the non-aquatic vectors as mature (*M*) adult mosquitoes. The parameter, *p*, represents the development of immature vectors to adult vectors. The parameters *μ*_*I*_ and *μ*_*M*_ are the mortality rates for the immature and mature vectors, respectively. In adverse weather, the adult vectors (*M*) are assumed to adapt and move into the dormant state *M*_*d*_. The movement (plasticity) of mosquitoes between the active and dormant compartments is modelled by the parameters *d* (adaptation) and *w* (reactivation). **b** The curves used to model vector rainfall-based fitness, adaptation and reactivation between the dry and the wet seasons

A mosquito completes the four stages of its life-cycle in two disparate habitats, the aquatic stages (eggs, Stage 1; larva, Stage 2; and pupa, Stage 3) in water and the adult stage (non-aquatic and disease transmitting mosquitoes, Stage 4) on land. To simplify this process but retain the dynamical effect of density-dependent larval mortality, we combined aquatic stages 1–3 in a single compartment. Following the model structure developed by White et al. [39], we assume that an adult female mosquito lays *F* eggs per unit time which hatch and develop into larvae, pupae, and eventually emerge as adult mosquitoes. Let *I* denote the aquatic vector population and *M* the non-aquatic vectors (female adult mosquitoes) (Fig. 1a). Aquatic vectors become adult mosquitoes at rate *p* and experience a mortality rate *μ*_*I*_ which depends on the total aquatic stages carrying capacity, *υ*. Assuming mosquitoes have an equal male-female sex ratio, a proportion, *p*/2, will be female. Adult mosquitoes are assumed to have a constant daily mortality rate *μ*_*M*_. Thus

1 $$ \frac{dI}{dt}= FM- pI-{\mu}_I\left[1+\frac{\upsilon I}{R(t)}\right]I,\kern0.5em \frac{dM}{dt}=\frac{pI}{2}-{\mu}_MM, $$

This model assumes the carrying capacity of the aquatic population scales linearly with *R*(*t*) , the 7-day moving average of rainfall. Under our null model, *M*_0_, we assume that mosquitoes will always lay a constant number, *F*, of eggs. Mosquito populations are forced to zero in the dry season when they fall below a given threshold (3.0e-7 and 1.0e-2 for adult and immature vectors, respectively), see Additional file 1: Text 1 for more details) to represent finite populations within a differential equation formulation.

We additionally consider the availability of rainfall (surface water) as a limiting factor in the dry season. Under this alternative model (*M*_1_), the mosquitoes’ ability to lay eggs when there is no surface water. We therefore introduce a ‘rainfall fitness function’, *f*_*R*_(*R*(*t*), *t*), to differentiate the number of eggs laid by each mosquito, *F*(*R*(*t*)) = *F*. *f*_*R*_(*R*(*t*), *t*) where *F* is a constant between the wet and the dry season. Breeding sites do not immediately become productive: there is a build-up time to gather enough water for mosquitoes to lay eggs. Also, during an interval of intermittent or no rainfall, they do not instantly dry up. We capture this effect using a logistic function

$$ {f}_R\left(R(t),t\right)=\frac{1}{1+{e}^{\left({m}_o-R(t)\right)}}. $$

The parameter *m*_*o*_ is the rainfall threshold that enables breeding sites to either become productive or stop being productive, respectively.

### Introducing aestivation mechanisms to capture dry season ecology

To extend our baseline mosquito population model to capture aestivation, we introduce a second class of aestivating vectors (Fig. 1a, b). Active adult vectors are assumed to aestivate at rate *d*(*R*(*t*)) that depends on recent rainfall, *R*(*t*). We further assume that aquatic stages do not aestivate (a reasonable assumption for *Anopheles* mosquitoes [9, 16, 19, 24]). We further assume that mosquitoes that fail to aestivate will not survive the dry season. Aestivating adult mosquitoes re-activate at a rate *ω*(*R*(*t*)) depending on recent rainfall *R*(*t*). The parameter *μ*_*d*_ is the mortality rate for adult aestivating mosquitoes. We also make a simplifying assumption that during the dry season mosquitoes do not lay eggs (or the contribution of eggs laid in the dry season to the mosquito population is negligible, since all the aquatic stages cannot survive without surface water). This is supported by studies that demonstrate mosquito reproduction arrest during the dry season [14, 15, 18] as well as those that suggest that gravid mosquitoes potentially sit out the dry season until the arrival of the rainy season to lay eggs or can resorb their eggs due to oviposition deprivation [16, 19, 24]. Our model including aestivation (termed model *M*_2_ ) is thus given by the following set of differential equations:

2 $$ \frac{d}{dt}=F\left(R(t)\right)M- pI-{\mu}_I\left[1+\frac{vI}{R(t)}\right]I,\frac{dM}{dt}=\frac{pl}{2}-{\mu}_MM-d\left(R(t)\right)M+\omega \left(R(t)\right){M}_d,\frac{dM_d}{dt}=d\left(R(t)\right)M-\omega \left(R(t)\right){M}_d-{\mu}_d{M}_d $$

where *M*_*d*_ denotes the aestivating adult mosquitoes.

### Phenotypic adaptation and reactivation fitness functions

Mosquito phenotypic plasticity parameters are multiplied by the functions *f*_*D*_ and *f*_*A*_, respectively. Therefore, the rainfall dependent and the mosquito active-dormant plasticity parameters are expressed as *F*(*R*(*t*)) = *F*. *f*_*R*_(*R*(*t*), *t*), *d*(*R*(*t*)) = *d*. *f*_*D*_(*R*(*t*), *t*) and *ω*(*R*(*t*)) = *ω*. *f*_*A*_(*R*(*t*)), where *f*_*D*_(*R*(*t*), *t*) and *f*_*A*_(*R*(*t*), *t*) are mosquito phenotypic adaptation and re-activation fitness functions.

The phenotypic adaptation of vectors into the dormant state is modelled using a Normal distribution function with mean, *m*_*o*_, as the optimal rainfall level for phenotypic adaptation (set at the same value as the threshold value for egg laying) and standard deviation, *d*_*v*_ = *m*_*o*_/4, as the interval within which phenotypic adaptation is viable. Phenotypic adaptation starts as rainfall levels begin to shrink, peaks when average rainfall reaches the value *m*_*o*_, after which it gradually declines. Thus:

$$ {f}_D\left(R(t),t\right)={N}_k\ \left({e}^{-0.5\frac{{\left(\ R(t)-{m}_o\right)}^2}{d_v^2}}\right),\mathrm{if}\ \frac{d{f}_R\left(R(t),t\right)}{dt}<0,\mathrm{otherwise}=0, $$

where *N*_*k*_ is a normalising constant.

Reactivation is modelled with a logistic function. Once reactivation is triggered mosquitoes will exit dormancy since there is no selective advantage for mosquitoes to retain their dry season traits. Thus:

$$ {f}_A\left(R(t),t\right)=1/\left(1+{e}^{2\left({m}_o-R(t)\right)}\right),\mathrm{if}\ \frac{d{f}_R\left(R(t),t\right)}{dt}>0,\mathrm{otherwise}=0. $$

The term 2(*m*_*o*_ − *R*(*t*)) simulates hesitance to exit dormancy as surface water builds up (when *R*(*t*) < *m*_*o*_) and rapid emergence as surface water accumulate to values greater than *m*_*o*_, *R*(*t*) > *m*_*o*_. The selection between the adaptation and reactivation is modelled using the gradient of the rainfall fitness function,$\frac{d{f}_R}{dt}$. If the gradient is negative, then adaptation is selected whilst a positive gradient will select for reactivation (Fig. 1b).

A full list of the model parameters and their description is included in Table 1.

**Table 1:** Model parameters and assumed values. Priors and fixed values were obtained from the literature

Parameter	Description	Units	Fitted	Fixed value/Uniform distribution for priors	Reference
*F*	Number of eggs laid	Per day	Yes	10 (2–25)	[39, 49–51]
*p*	Aquatic vectors maturation rate	Per day	No	0.07	[39, 49–51]
*μ*_*I*_	Aquatic vectors mortality rate	Per day	No	0.3	[39, 49–51]
1/*μ*_*M*_	Adult vectors mortality rate	Days	Yes	12.0 (7.0–21.0)	[39, 49–51]
1/*μ*_*d*_	Aestivating vectors mortality rate	Days	Yes	100.0 (21.0–217.0)	[3, 4]
*υ*	Rainfall carrying capacity scale factor	Constant	No	17.0	[39]
*d*	Adult mosquito adaptation to dormancy	Per day	Yes	0–1	–
*ω*	Exit of adult mosquitoes from dormancy	Per day	Yes	0–1	–
*m*_*o*_	Optimal rainfall/surface water	Constant	Yes	25–100	–

### Longitudinal data on mosquito populations

Time series adult mosquito count data were digitized from the study by Adamou et al. [3] who recorded mosquito populations between two wet seasons in Mali. The study was carried out between September 2009 and October 2010 and accounts for both dry season and wet season vector population dynamics. During the study period surface water and rainfall were absent for most of the 7-month dry season. The study paired villages together based on their proximity and randomly assigned one of the villages as a control and the other as treated. Treatment involved weekly pyrethrum spraying in all houses in each village throughout the dry season. Pyrethrum spraying was undertaken to eliminate aestivators in the treatment arm only whilst the selection of distant villages was done to minimize the effect of mosquito migration between villages so as to observe the contribution of aestivation to vector population build-up at the onset of the rainy season. Vector population dynamics were monitored for a period of approximately 14 months. We used the mosquito population data from the control villages to fit our models, with the vector species *A. coluzzii* and *A. arabiensis* analysed separately.

We additionally compared our fitted models to mosquito abundance data from Omer & Cloudsley-Thompson [9] who monitored vector populations in an area with a 9-month dry season and in a region where water persists throughout the year. We fitted the model separately for the dry arid region and a region with persistent water sources (the Nile Valley) to data for *A. gambiae* (*s.l.*) combined using ovarian arrest as a cue for aestivation. Model comparisons with these data are given in Additional file 1: Text 2 and Figure S2.

For both studies average rainfall data in the locations was obtained from the Climate Change Knowledge Portal (http://sdwebx.worldbank.org/climateportal/). For model fitting the 7-day moving average data were used.

### Model fitting

We fitted the model to the mosquito time series monthly count data under a Poisson likelihood using an MCMC algorithm implemented in R using the FME package [44]. Parameter posterior distributions were drawn from 100,000 MCMC samples discarding a burn-in period, assuming Uniform priors for all fitted parameters (Table 2 and Additional file 1: Table S1). The chains were analysed visually for convergence. Parameters were estimated as the median of the posterior sample. The 50% and 95% credible intervals for the estimated parameters were computed using the 25–75 and 2.5–97.5 quantiles of the MCMC chain. A sensitivity analysis to the fixed parameters is provided in Additional file 1: Figures S3 and S4. Model fits were compared using the deviance information criterion (DIC), Table 2.

**Table 2:** Posterior estimates of parameters and model fit. The 95% credible intervals are given in parentheses. Other parameters were fixed during model fitting at the values given in Table 1. DIC denotes the deviance for each fitted model with the lowest value representing the most parsimonious model. DIC values in bold identify the best model hypothesis that recapitulates the data

Model variant	*F*	1/*μ*_*M*_	1/*μ*_*d*_	*d*	*ω*	*m*_*o*_	DIC
*Anopheles coluzzii*							
H:0	1.4 (1.1–1.7)	38.1 (35.8–42.5)	–	–	–	–	166.0
H:1	1.6 (1.2–2.0)	56.0 (46.6–67.5)	–	–	–	73.4 (70.1–89.5)	216.2
H:2	3.3 (2.7–3.9)	19.8 (18.7–20.0)	155.5 (120–198)	0.011 (0.010–0.017)	0.95 (0.74–1.0)	75.4 (70.0–80.0)	248.9
H:3	7.5 (4.9–9.3)	10.9 (10.0–14.5)	114.5 (71–174)	0.012 (0.010–0.030)	0.76 (0.51–0.99)	89.9 (72.7–99.5)	**119.8**
*Anopheles arabiensis*							
H:0	1.3 (1.0–1.8)	32.7 (25.5–39.7)	–	–	–	–	50.7
H:1	0.8 (0.6–1.0)	55.0 (46.7–64.3)	–	–	–	85.5 (77.7–92.6)	**41.8**
H:2	1.4 (1.0–2.0)	19.4 (15.4–20.0)	79.6 (43.4–162.5)	0.015 (0.010–0.040)	0.75 (0.51–0.99)	73.8 (70.1–93.6)	70.4
H:3	1.8 (1.0–2.6)	12.9 (10.1–18.9)	74.4 (46.6–152.7)	0.052 (0.010–0.100)	0.79 (0.52–0.98)	85.1 (70.8–99.2)	**39.4**

In the study by Adamou et al. [3] mosquitoes were caught indoors, making it impossible to differentiate quiescent indoor resting mosquitoes from aestivating mosquitoes. We therefore considered a number of different hypotheses to relate the model to the observed data: (i) H:0, all caught mosquitoes are non-aestivating (model *M*_0_); (ii) H:1, all caught mosquitoes are non-aestivating with reduced mosquito reproduction in the dry season (model *M*_1_); (iii) H:2, aestivation occurs but only non-aestivating mosquitoes are observed in the dry season (model *M*_2_); and (iv) H:3, mosquitoes caught in the dry season are both non-aestivating and aestivating (model *M*_2_).

### Estimating mosquito wet and dry season reproduction

We estimated the mosquito net reproduction,${\mathcal{R}}_0$ of the two models (without and with aestivation) using the next generation matrix method under the posterior median parameter sets. The net reproduction numbers are given by:

$$ {\mathcal{R}}_A=\frac{pF}{2{\mu}_m\left(p+{\mu}_I\right)} $$

$$ {\mathcal{R}}_D=\frac{pF}{2\left({\mu}_m+\frac{\mu_dd}{\left({\mu}_d+\omega \right)}\ \right)\left(p+{\mu}_I\right)} $$

where ${\mathcal{R}}_A$ is the net reproduction number in the model without aestivation and ${\mathcal{R}}_D$ the net reproduction number in the model with aestivation. The effective net reproduction numbers are given in Additional file 1: Figure S5.

### Modelling vector control interventions

Rather than explicitly modelling current vector interventions [39, 45], we considered the impact of tools that target the adult mosquito vector or aquatic stages and how dry season ecology could affect the success of these interventions (see Additional file 1: Text 1 for details on how interventions were simulated). We therefore consider increasing the mortality of adult vectors to represent insecticides targeting adult females or reducing the maturation rate of aquatic vectors as a proxy for larviciding. Thus, insecticides can target either active mosquitoes (during the rainy season), aestivating mosquitoes (during the dry season) or both (throughout the year). Larval control acts on all larvae as we consider aestivating and non-aestivating mosquitoes to be phenotypic variations of the same underlying population. We assumed both aestivating and non-aestivating mosquitoes to be equally susceptible to insecticides. This assumption is made given a lack of experimental evidence to support alternative hypotheses, though it is possible that aestivating vectors might be more resistant because of their altered physiology which makes them more stress tolerant.

Interventions are applied by increasing the larval and adult death rates respectively throughout the year and consecutively for 3 years and are then relaxed in the 4th year. The impact of each intervention is assessed by enumerating population reduction/suppression of active adult mosquitoes during the course of the intervention and afterwards.

## Results

**Fig. 2:**
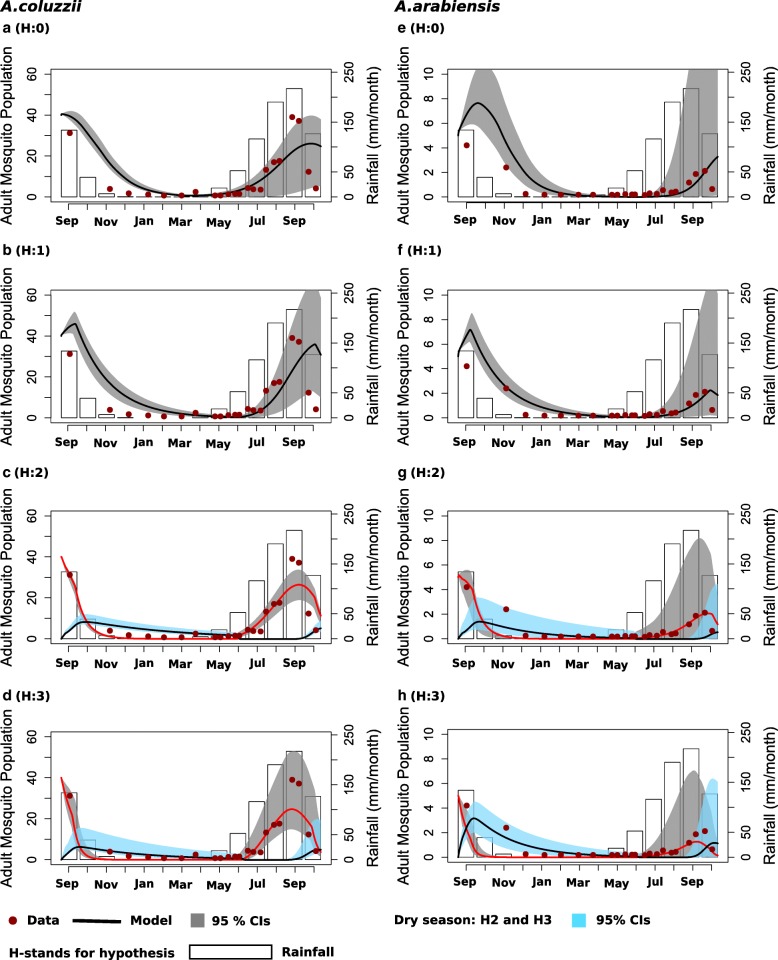
Model fits to the dry and wet season mosquito population data. Panels **a**-**d** show results for *A. coluzzii* and panels **e-h** for *A. arabiensis*. The null model H:0/*M*_0_ assumes no aestivation and no reduced fecundity in the dry season. Model H:1/*M*_1_ assumes no aestivation but reduced reproduction fitness in the dry season. Model H:2/*M*_2_ includes aestivation but that only the active vectors are observed. Model H:3/*M*_2_ assumes that both active and aestivating vectors are observed

**Fig. 3:**
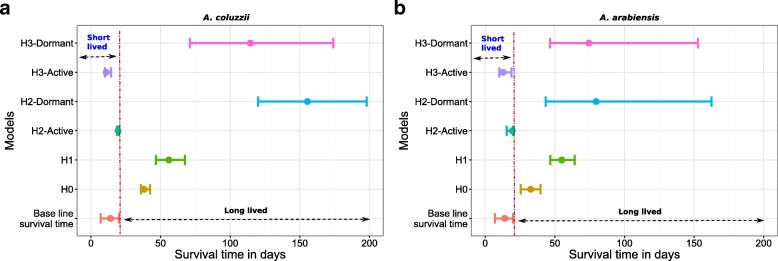
Predicted adult mosquito lifespans. H:0 and H:1 predict non-aestivating mosquitoes to be long-lived while the aestivation models (H:2 and H:3) predict only aestivating mosquitoes to be long-lived. The error bars represent the credible intervals and the dots the median of the estimated parameters. Panels **a** and **b** show the predicted lifespan for *A. coluzzii* and *A. arabiensis* adult mosquitoes

Figure 2 shows the fit of the models to the data from Adamou et al. [3] whilst the estimated model parameters and DIC values for the model fits are shown in Table 2. For *A. coluzzii*, the model with aestivation included under the assumption that the data represent both aestivating and non-aestivating vectors (H:3) provides the best fit to the data (Fig. 2, Table 2**)**. Furthermore, this model gives estimates of lifespan for active mosquitoes consistent with other studies (median survival time of 10.9 days, 95% credible interval, CrI: 10.0–14.5 days, Table 2, Fig. 3) alongside estimates of longer median survival in aestivating mosquitoes (median survival time of 114.5 days, 95% CrI: 71.0–174.0 days). The DIC values indicate that the model with no aestivation (H:0) provides the second-best fit to the *A. coluzzii* data but in doing so estimates a long lifetime for mosquitoes in order to survive the dry season (median survival time of 38.1 days, 95% CrI: 35.8–42.5 days) (Table 2, Fig. 3). For *A. arabiensis*, a similar pattern emerges, with the same model (H:3) providing the best fit to the data as judged by the DIC. However, there is little difference in the DIC score between models H:3 and H:0 (no aestivation) suggesting that a model without aestivation can capture the observed data adequately (Fig. 2, Table 2). However, as in the fits for *A. coluzzii*, the median survival time estimates for *A. arabiensis* under model H:0 are long (median survival time of 32.7 days, 95% CrI: 25.5–39.7 days). Similar results were obtained using village paired mosquito population data and data from Sudan (Additional file 1: Figures S1 and S2, Table S2).

**Fig. 4:**
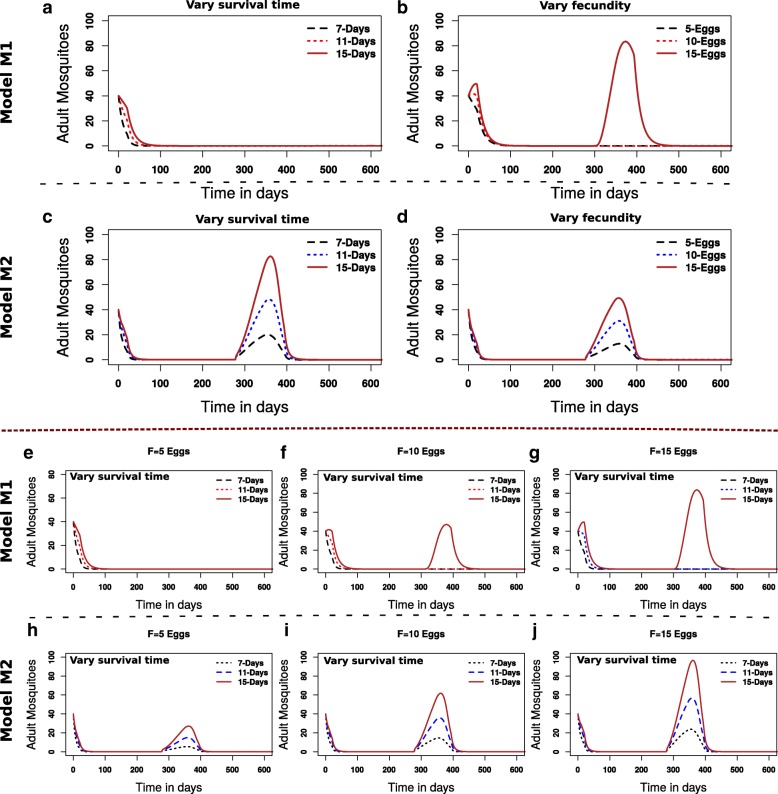
The trade-off between fecundity and survival longevity drives population persistence. *A. coluzzii* mosquito populations are shown to go extinct if fecundity and survival times are reduced. Panel **a** illustrates extinction (in model *M*_1_), survival time is increased while keeping fecundity low (F = 5 eggs). Panel **b** shows that persistence is achieved be increasing F to 15 eggs. Panels **c** and **d** demonstrate that persistence is easily sustained in the aestivation model, *M*_2_, at low active adult mosquito survival times and minimal reproduction. Panels **e-g** show the trade-off between fecundity and survival longevity in the no-aestivation model. Increasing fecundity (eggs laid), allows relatively short-lived mosquitoes to persist. Panels **h-j**, demonstrate more robust persistence under similar conditions in the aestivation model. Parameters used are as given in Tables 1 and 2; however, the parameters F (fecundity) and *μ*_*I*_ or *μ*_*d*_ (survival) are varied as shown in the panels

Figure 4 shows the predicted mosquito population persistence dynamics under the different models. Under the model with no aestivation and with low fecundity during the dry season (hypothesis H:1, model M1), if fecundity is low in the rainy season then persistence cannot occur even for a range of plausible survival times (Fig. 4a). In this model, sustained populations can only be achieved if fecundity in the rainy season is assumed to be higher and/or survival times are longer (Fig. 4b, e-g)**.** In contrast, by explicitly including aestivation in the model (model M2, hypotheses H:2 and H:3, Fig. 4c, d), persistence can occur over a wider range of both parameters (i.e. shorter survival times and lower fecundity, Fig. 4h-j)**.**

We undertook an additional sensitivity analysis to ascertain how the model parameters that were fixed during model fitting influence the population of active and aestivating mosquitoes (see more details in Additional file 1: Figure S4). The level of rainfall during the rainy season is positively correlated with the size of the active mosquito population, whilst the level of rainfall during the dry season is negatively correlated with the aestivating mosquito population. Populations of both active and aestivating mosquitoes are also sensitive to the level of rainfall required to determine phenotypic adaptation.

**Fig. 5:**
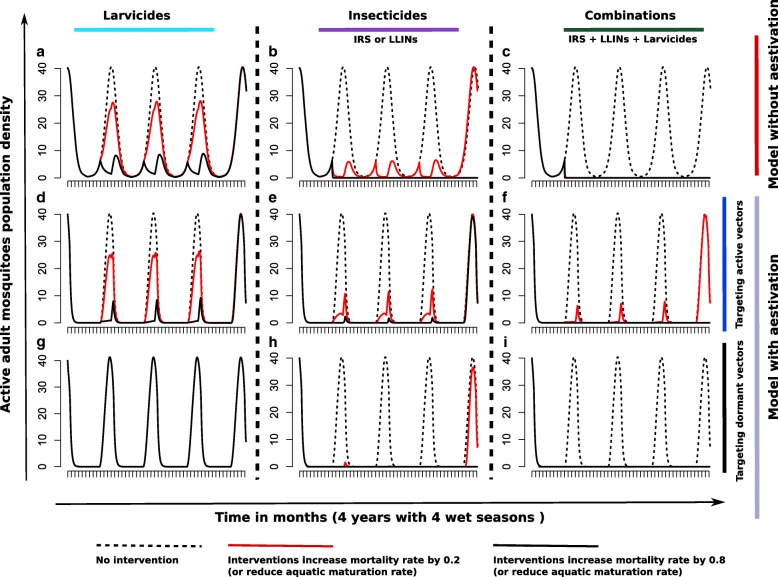
Evaluation of vector control strategies. Simulations showing the modelled changes in the abundance of active adult vectors when interventions with different mechanisms of action are used to control the mosquito population. Panels **a**, **d** and **g** show the effects of interventions that increase the larval death rate (e.g. larvicides) whilst panels **b**, **e** and **h** show the effects of interventions that kill adult mosquitoes (e.g. IRS, LLINs or other insecticides). Panels **c**, **f** and **i** show the effects of combining these two targets. Panels **a**-**c** are predicted from a model with no aestivation; **d-f** from a model including aestivation in which only the active vectors are targeted and **g-i** from the aestivation model if dormant vectors are also targeted. Parameter values used are given in Tables 1 and 2. Intervention efficacies of 0, 20 and 80% were used, and these represent percentage increase in mortality of vectors/larvae induced. The dotted line (0%) means no intervention; the red line and the black line represent interventions that increase vector/larvae mortality by 20 and 80%, respectively. See Additional file 1: Text 1 for further details on how interventions were simulated

We next explored the effect of reducing either the aquatic stage survival or adult vector survival under the different models. Figure 5 shows the predicted impact of these two strategies, alone and in combination, on the vector population. In the models with no aestivation, both strategies are predicted to dramatically reduce vector populations, although this rebounds if the effect is not sustained and the vector population is not eliminated (Fig. 5a-c). Additionally, reducing aquatic stage survival alone is not predicted to be as effective as targeting adult mosquitoes in this model (Fig. 5a, d, g). In the model with aestivation, we predict a lesser impact of vector control if only the active vectors are targeted since here the aestivating vectors always allow re-emergence of the vector population once the intervention ceases (Fig. 5e, f). However, if the aestivating vectors are additionally targeted during the dry season (as dormant vectors), a greater impact is predicted with a higher probability of extinction (Fig. 5h, i).

## Discussion

Extensive study of the dry season ecology of malaria vectors [3–5, 8, 14, 15, 24] suggests that the *Anopheles* mosquitoes evade population extinction by aestivating locally or by migrating to locations where water persists. We explored the first of these two mechanisms to understand (i) whether aestivation alone could explain the observed persistence and (ii) the implications of this for vector control. By fitting our model to data from two seasonal areas of Africa, we have shown that an aestivation mechanism whereby mosquitoes adapt their behaviour between the wet and the dry seasons is able to reproduce observed patterns, hence providing a plausible explanation for the re-emergence of mosquitoes at the end of the dry season. Insects are known to be able to adapt to ecological and environmental alterations to avoid extinction [18, 30–32]. In general, different organisms can resist the effects of environmental change by behavioural changes, migration and physiological acclimation (phenotypic plasticity), thereby facilitating persistence of otherwise non-persistent species. Phenotypic plasticity therefore likely enables *Anopheles* mosquitoes to survive cyclic environmental changes.

Given that the wet season in the African Sahel is short-lived compared to the dry season, fast and high reproductive output during the wet season is one mechanism that could ensure rapid mosquito population expansion within a short time as observed in the data used here and in several other studies [9, 11, 14, 29, 46, 47]. Whilst this can help protect against extinction, our modelling results suggest that a large population in itself is not sufficient, since for a single location model (i.e. ignoring migration) in which there is no aestivation mechanism, we cannot reproduce the observed data unless biologically unrealistic long durations of mosquito longevity are assumed. This observation suggests that there are two selection bottlenecks that drive phenotypic plasticity. The first occurs at the beginning of a dry season and selects for mosquitoes that can survive the long dry stretch and the second at the onset of a new wet season, when mosquitoes must exit diapause or dormancy to resume rapid reproduction. This rapidly changing environment, including variation year-on-year in the timing of the seasons, implies that offspring will face different conditions from their parents, supporting a phenotypic switch rather than inheritance [16, 19, 20, 22, 31, 32].

Phenotypic plasticity has clear consequences for interventions that aim to reduce the adult female mosquito population responsible for malaria transmission. Our results demonstrate that, if aestivation is present, vector-based interventions targeting aestivating mosquitoes in addition to active mosquitoes could further increase the overall effectiveness of vector control. Such a strategy could involve the use of insecticides during the dry season to reduce the aestivating mosquito population through application to areas in which they are known to hibernate. However, identifying places were aestivating mosquitoes hide or hibernate is inevitably challenging and likely to differ in different ecological settings and hence require further research. Furthermore the potential additional effectiveness of this strategy alongside existing vector control efforts (LLINs, IRS and larviciding) would require localised testing. Such strategies could, however, be of particular relevance to malaria control and elimination efforts in the African Sahel region, where mosquitoes have been observed to survive the long dry season and vector control remains challenging.

An additional (or alternative) mechanism that could explain the persistence of *Anopheles* vectors during the dry season is migration [5]. In a recent modelling study, North & Godfray [48] considered the combined role of migration and aestivation using a simulation-based approach applied to Burkina Faso. Their results showed that, whilst local dispersal could explain the spatial distribution of mosquito populations in most areas, it failed to capture mosquito populations in the very dry areas of the northern Sahel region. Aestivation could explain seasonal population dynamics in much of the region, but some long-distance migration was necessary to capture all populations. This study took a different modelling approach to that adopted here, namely using data to obtain parameters that are entered into a more complex simulation-based framework rather than statistically fitting a simpler model to data. That they come to similar conclusions regarding a potential role for aestivation is therefore reassuring. An alternative approach to address the relative roles of migration and aestivation would be to collate population genetics data for vector species in space and in time. Such data could help to understand the extent to which emerging mosquito populations are genetically distant from the previous season populations.

There are a number of further limitations to the approach taken here. First, to enable robust model fitting, we explored a limited set of relatively simple model structures. In further work, it would be possible to expand the model to consider other mechanisms that prompt aestivation, for example by considering alternative parametric forms as well as additional environmental triggers. The latter is clearly important to explain the increase in mosquito populations observed by Adamou et al. [3] that occurred prior to the onset of the rains. The modelling framework can also be extended into a spatial meta-population framework to allow an assessment of the relative roles of migration and aestivation. However, in doing so, it is important to capture the relative densities of mosquito populations at a range of spatial scales. Secondly, we considered the potential implications for vector control in a relatively simplistic way by simply increasing the death rate of either the adult or larval populations. In other work we have explored the impact of more detailed models that capture the full mode of action (both killing and repellence) of interventions such as LLINs and IRS [39, 45]. These more complex models are important when considering the impact of interventions on human endpoints because of the community benefits of vector control.

Our results also raise some practical challenges for ecological field studies. First, it remains unclear what populations are captured during the dry season in field studies. Mosquito catch traps are normally installed in human houses or outside human dwelling places since mosquitoes are drawn to them in their search for blood meals. However, it is unknown whether aestivating mosquitoes are likely to be found in these locations and hence it is likely that this presents a partial picture. Secondly, the longevity of individual mosquitoes remains uncertain. One study [4] observed a single mosquito that survived for approximately seven months in a mark-release-recapture field experiment, which might represent the upper boundary of mosquito dry season survival. Our results suggest however that persistence can be achieved with lower average longevity for aestivating mosquitoes (median of 114.5 days for *A. coluzzii* compared to 217 days (7 months) observed in study [4]). Further mark-capture-recapture studies coupled with sequencing of the *Anopheles* genome from the wider region could help to further elucidate the balance between longevity, reproduction and migration.

In summary, whilst our model illustrates the potential for aestivation to explain the dry season persistence of *A. coluzzii* mosquitoes, much remains to be understood. Nevertheless, our results illustrate that it is critical that more efforts are directed towards understanding the dry season ecology of mosquitoes as a mechanism for mosquito persistence if efforts to control and eliminate malaria, particularly in the Sahel region of Africa, are to be successful.

## Conclusions

Overall, our study demonstrates that the dry season ecology of *A. coluzzii* mosquitoes can be explained by the aestivation phenomenon. However, for *A. arabiensis* mosquitoes, dry season survival longevity is not a requirement for their persistence. Which suggests that other strategies (such as migration) could be at play. Our analysis of vector control interventions shows that the ability of *A. coluzzii* to persist in the dry season calls for innovative control strategies that specifically target aestivating mosquitoes for increased prospects of eliminating malaria in the Sahel region.

## Additional file

Additional file 1: **Text 1.** Additional Methods. **Text 2.** Additional model fits. **Figure S1.** Fitting models to the dry and wet season mosquito population data from two villages. **Figure S2.** Fitting models to the dry and wet season mosquito population data from Sudan. **Figure S3.** The effect of dry season minimum adult mosquito populations on estimates of the adult survival time in a model with no aestivation. **Figure S4.** Sensitivity analysis of model parameters. **Figure S5.** Mosquito seasonal net reproduction numbers. **Table S1.** Estimated parameters by fitting village pair data. **Table S2.** Estimated parameters using data from Sudan. (DOCX 4304 kb)

## Abbreviations

DIC: Deviance information criterion
CrI: Credible intervals
*s.s.*: *sensu stricto*
*s.l.*: *sensu lato*
MCMC: Markov Chain Monte Carlo
