# Supplementary material for: A trade-off between dry season survival longevity and wet season high net reproduction can explain the persistence of Anopheles mosquitoes
Source: Parasit Vectors. 2018 Nov 3;11:576. doi: 10.1186/s13071-018-3158-0 (PMC6215619; doi:10.1186/s13071-018-3158-0)
Supplement: Supplementary file 1 — Text 1. Additional Methods. Text 2. Additional model fits. Figure S1. Fitting models to the dry and wet season mosquito population data from two villages. Figure S2. Fitting models to the dry and wet season mosquito population data from Sudan. Figure S3. The effect of dry season minimum adult mosquito populations on estimates of the adult survival time in a model with no aestivation. Figure S4. Sensitivity analysis of model parameters. Figure S5. Mosquito seasonal net reproduction numbers. Table S1. Estimated parameters by fitting village pair data. Table S2. Estimated parameters using data from Sudan. (DOCX 4304 kb) [file 13071_2018_3158_MOESM1_ESM.docx]

**A trade-off between dry season survival longevity and wet season high net reproduction can explain the persistence of *Anopheles* mosquitoes**

**Gesham Magombedze^1,2^, Neil M Ferguson^2^ and Azra C Ghani^2^**

1. Center for Infectious Diseases Research and Experimental Therapeutics, Baylor Research Institute, Baylor University Medical Center, Dallas, TX, USA.
2. MRC Centre for Global Infectious Disease Analysis, Department of Infectious Disease Epidemiology, Imperial College London.

**Supplementary Information: Additional file 1**

# Additional Methods

## Dry season mosquito population population limits

We set a cap of 3.0E-7 (values less than this value were set to zero) on the adult mosquito population to avoid simulating infinitesimally small numbers. We make use of mosquito estimates from a capture mark release study [1]. The studies estimated 12670 mosquitoes per 275 people in Mali during the rainy season or 46 mosquitoes per person. This maps to ~38 mosquitoes per person in the study of Adamou et al. [2] since 10602 female mosquitoes were caught. Estimating the human population to be 2.42 million in the region, the threshold below which extinction occurs is in the study of Adamou is calculated to be $\frac{38}{46\times\left( 2.43E6 \right)}=$3.4E-7 which is ~3.0E-7.

## Deviance Information Criterion

We used the Deviance Information Criterion (DIC) to select the model and the fitting hypothesis that best explains the data. A model with a small DIC value is preferred. A difference of less than 2 in the DIC values between models represent insufficient information to reject a model or select one model above the other. DIC differences larger than 10 rule out the model with a larger DIC, while DIC values between 3 and 10 suggest moderate evidence to prefer a model with a small DIC value.

$$DIC=\hat{D}+pD$$

Where $pD=\hat{D}-D(\hat{\theta})$, $D=-2logL(y/\theta)$ and $y$ is the data, $\theta$ the paramerers and $L$ is the model likelihood.

## The effective net reproduction number

The effective reproduction number quantifies mosquito reproductive potential in the presence of density dependent mortality (unlike the basic reproduction number which applies in the low density limit). It is defined as

$$\mathcal{R}_{Ae}=\frac{pF}{2\mu_{m}(p+\mu_{I}(1+\nu I(t)/R(t)))},$$

$$\mathcal{R}_{De=}\frac{Fp}{2\left( p+\mu_{I}(1+\nu I(t)/R(t)) \right)\left( \mu_{m}+\frac{d\mu_{d}}{\mu_{d}+\omega} \right)}.$$

Here $I(t)$ is the size of the larval population at time *t*, and $R(t)$ is the 7-day average of rainfall. Effective net reproduction throughout the wet and dry seasons is shown with simulations in Fig. S5**.**

## Evaluating the impact of interventions

As described briefly in the main text, we used a simplified modification to capture the key effects of vector control intervention strategies. Three different vector control strategies are explored:

1. Larvicides and insecticides are administered in the rainy season for a period of six months (starting 2 weeks before the onset of the rainy season). We make a simplifying assumption that insecticides will increase the mortality of adult mosquitoes (effectively reduce their survival time) with a specific percentage (0%, 10%, and 80%). We assume that larvicides will reduce the viability of breeding sites through reducing the carrying capacity and reducing the maturation probability of aquatic vector by 0%, 10%, and 80%**.** Because this intervention is only applied during the rainy season, in our model the insecticides will predominantly target non-aestivating mosquitoes.
2. Based on the aestivation model assumption, we simulate and evaluate the theoretical impact of insecticides that only target and kill dormant adult mosquitoes in hibernation (and thus would represent an intervention implemented during the dry season. The study by Omer and Cloudsley-Thompson, [3], prove the existence of several hibernation sites that are potential places to apply insecticides.
3. The wet season and dry season interventions are combined. In all the strategies, the potency of each strategy is evaluated by quantifying rebounding active adult mosquito populations after 3 years of continuous vector control.

# Additional Model Fits

## Fits to the separate village data

In the study of Adamou et al. [2], mosquito populations were measured in two different village pairs. In the main manuscript we fitted our models to the combined adult mosquito population data. We additionally tested the robustness of this approach by fitting the model jointly to the two separate village populations Fig. S1. In doing so, we estimate relatively similar vector life history parameters compared to fitting the combined data, Tables 1, 2 and S1.

**Figure S1: Fitting models to the dry and wet season mosquito population data from two villages.** Panels **A**, **B, C** and **D**, show model fitting results to the *A. coluzzii* vector species. Results for fitting the *A. arabiensis* adult mosquito population are shown in panels **E, F, G** and **H**. Data between the two villages were fitted jointly under hypotheses **H:0**, **H:1, H:2** and **H:3**.

**Table S1**: Estimated parameters by fitting village pair data. The parameters were estimated using three different hypotheses **H:0**, **H:1**, **H:2** and **H:3**. The DIC is used to select the model that best explains the data. Parameters that were keep fixed during fitting are given in Table S1. The uncertainty of the estimated parameters is shown by the 95% credible intervals that are given in brackets.

| Model | $F$ | ${1/\mu}_{M}$ | $1/\mu_{d}$ | $d$ | $\omega$ | $m_{o}$ | DIC |
| --- | --- | --- | --- | --- | --- | --- | --- |
| *Anopheles coluzzii* | | | | | | | |
| H:0 | 1.51 (1.3,1.7) | **32.56 (31.1, 35.7)** | - | - | - | - | 345.4 |
| H:1 | 1.14 (1.0,1.40) | **64.0 (56.0, 72.4)** | - | - | - | 72.09 (70.1, 88.0) | 471.6 |
| H:2 | 2.86 (2.4,3.4) | 19.86 (19.1, 20.0) | 113.4 (86.8, 149.8) | 0.022 (0.02, 0.023) | 0.96 (0.84, 1.0) | 71.51 (70.2, 72.6) | 492.9 |
| H:3 | 5.10 (4.3,6.6) | 13.26 (11.6, 14.4) | 120.4 (81.3, 157.5) | 0.013 (0.01, 0.024) | 0.90 (0.69, 1.0) | 96.0 (72.7, 99.8) | **265.4** |
| *Anopheles arabiensis* | | | | | | | |
| H:0 | 1.09 (1.0,1.5) | **38.20 (30.6, 43.7)** | - | - | - | - | 121.3 |
| H:1 | 1.04 (1.0,1.2) | **49.16 (41.6, 56.6)** | - | - | - | 89.2(70.8,99.6) | 108.9 |
| H:2 | 1.51 (1.2,1.8) | 19.40 (15.2, 20.0) | 70.26(54.8,97.9) | 0.015(0.01,0.03) | 0.80(0.51,0.98) | 71.50(70.1,88.5) | 149.5 |
| H:3 | 1.75 (1.2,2.2) | 12.26 (10.1, 18.0) | 94.35(69.0,160.1) | 0.086(0.02,0.19) | 0.84(0.71,0.99) | 85.09(71.6,98.5) | **88.97** |

## Testing the model with a different aestivation data set

To further test our models and the robustness of our predictions we used a different data set [3]. This study was carried out in Sudan where two mosquito populations were monitored over a period of 14 months (including a 9 month dry period) in two different areas in Khartoum region. The study found out that a member of the *A. gambiae* complex species maintained itself by low-level breeding along the White Nile valley. This was proved by the continual presence of larvae and parous females throughout the dry months. However, in the second area, an arid region (Fattasha region) situated more than 20 km from the Nile valley, no parous *A. gambiae* female adult mosquitoes were detected. Also, no breeding was evident. In this area, adult mosquitoes were found in occupied huts, deserted huts, dry wells and animal burrows. During the dry season, female mosquitoes in this area failed to complete the development of all their ovarian stages. However, parous mosquitoes were detected after the rainy season resumed.

To fit the model along the Nile valley, we imposed a constant source of surface water to simulate the persistence of breeding sites throughout the year. In our model fitting we used the population of parous adult mosquitoes since they represent mosquitoes that were active (and not aestivating). We test hypotheses H:0 and H:3.

The results are shown in Fig. S2 and are consistent with our findings shown in Fig. 2 and S1 obtained using data from Mali.

**Figure S2: Fitting models to the dry and wet season mosquito population data from Sudan.** Panels **A**, **B, C** and **D**, show model fitting results to the *A. gambiae* complex vector species observed in the study of Omer and Cloudsley-Thompson [3] between two separate regions. Panels **A** and **B** show parous female mosquitoes in the arid region of Fattasha, while Panels **C** and **D** show parous adult mosquitoes observed along the Nile valley. Models **H:0**and **H:3** were used to explain the vector population dynamics between these two regions. Estimated parameters are in Table S3**.**

**Table S2**: Estimated parameters using data from Sudan [3]. The uncertainty of the estimated parameters is shown by the 95% credible intervals. Parameters that were not varied during model fitting are as given in Table S1. DIC is the deviance for each model used and the model with the least DIC value is preferred.

| Model | $F$ | ${1/\mu}_{M}$ | $1/\mu_{d}$ | $d$ | $\omega$ | $m_{o}$ | $K_{c}$ | DIC |
| --- | --- | --- | --- | --- | --- | --- | --- | --- |
| Fattasha Region | | | | | | | | |
| H:0 | 16.8 (9.5,19.9) | **24.2 (20.4, 29.3)** | - | - | - | - | 7.20  (3.8, 7.2) | -4158 |
| H:3 | 14.8 (10.9, 19.3) | 10.5 (10.0, 12.8) | 138.6 (108.0, 179.8) | 0.018 (0.01, 0.03) | 0.21 (0.01, 0.72) | 26.5 (16.4, 29.9) | **1.16 (1.0, 1.9)** | -4525 |
| Nile Region | | | | | | | | |
| H:0 | 12.2 (7.5,14.6) | **11.2 (10.1, 13.6)** | - | - | - | - | 8.7  (4.6, 13.8) | -1196 |
| H:3 | 3.5 (2.7,4.9) | 10.8 (10.0, 12.8) | 46.7 (40.3, 194.0) | 0.021 (0.01, 0.06) | 0.88 (0.65, 0.99) | 25.0 (24.4, 25.0) | **1.29**  **(1.0, 3.1)** | **-1259** |

# Sensitivity Analyses

## Minimum adult population threshold

Fig. S3 shows the sensitivity of our results to different assumptions regarding the minimum adult mosquito population size in the dry season. This demonstrates that deterministic models in which aestivation is not included can only capture seasonal dynamics by either allowing small populations that continue to breed or by increasing survival times.


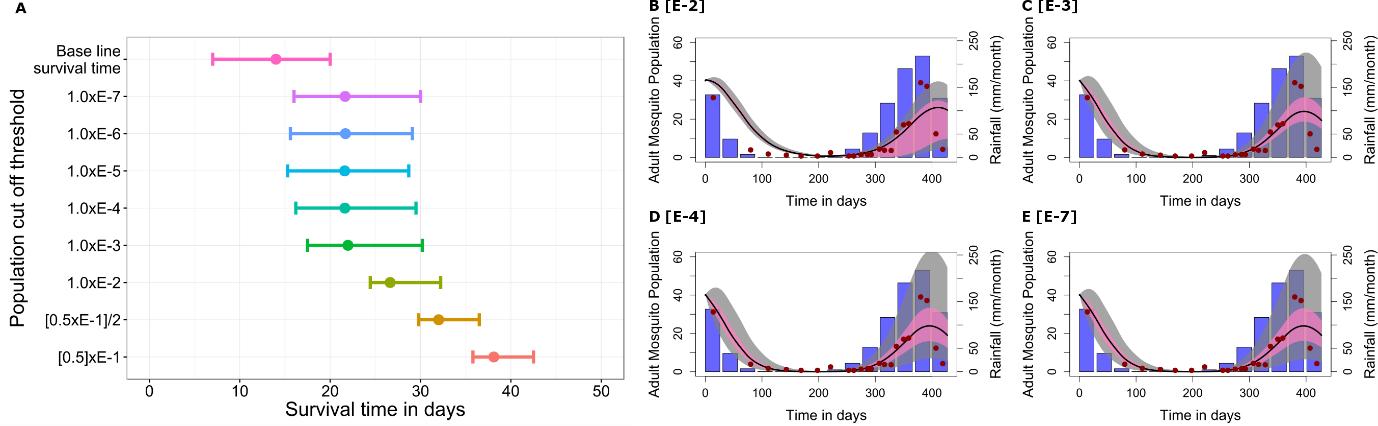


**Figure S3**: **The effect of dry season minimum adult mosquito populations on estimates of the adult survival time in a model with no aestivation**. The baseline estimate is that obtained with no population threshold (i.e. allowing infinitesimal population sizes). As the threshold is increased, the estimated survival time for adult mosquitoes increases in order to sustain the population through the dry season.

## Fixed parameter sensitivity analysis

We undertook a sensitivity analysis to ascertain how the parameters that were fixed during model fitting influence two key model outputs - the population of active and dormant mosquitoes in the dry and rainy seasons. Latin hypercube sampling (LHS) was used to generate parameter samples. We used the partial rank correlation coefficients (PRCCs) as a qualitative measure to indicate how a change in the value of a given parameter will cause a change in the mosquito population. A negative PRCC value indicates that increasing the corresponding parameter will result in areduction in the predicted population size whilst a positive value indicates that increasing the parameter will result in an increase in the population size. The magnitude of the PRCC, which is between -1 and 1, is a quantitative predictor of how strongly the parameter alters the model output variables.

Fig. S4 shows the PRCC values for five model parameters for adult active mosquitoes (panel A) and adult aestivating mosquitoes (panel B). The aquatic vector mortality rate is consistently negatively correlated, so that increasing the mortality rate decreases both adult populations as would be expected. Similarly, the aquatic vector maturation parameter has a strong positive correlation with the active mosquito population and for the aestivating mosquito population during the dry season, confirming that this parameter determines the growth of both the active and aestivating mosquitoes.

The rainfall carrying capacity is positively correlated with active adult mosquitoes throughout the transmission season, so that higher rainfall is predicted to increase the active adult mosquito population (with a lower correlation during the dry season compared to the rainy season). It is however negatively correlated with the aestivating mosquito population during the dry season because an increase in rainfall at this time would result in a phenotypic switch from aestivating to active mosquitoes.

The optimal rainfall signalling a switch to active mosquitoes from the dormant state has a correlated with the active mosquito population and positively associated with the dormant population. This implies that a high threshold value prevents early reactivation of active mosquitoes and therefore delays aestivating mosquitoes from exiting dormancy.

The rainfall history interval $t_{d}$ is not significantly correlated with the population of aestivating mosquitoes. However, it is positively correlated with the population of active mosquitoes since a longer interval results in a larger expansion of the population for a given rainfall dataset.

In general, the way these parameters influence the modelled vector population dynamics changes between the wet and the dry seasons. The parameters$\nu$,$\mu_{I}$ and $m_{o}$ significantly reduce both the dry and wet season vector populations and exert an opposing effect between the active and aestivation mosquito populations. Only parameter $p$ has a positive effect on both populations.

**Figure S4: Sensitivity analysis of model parameters.** The PRCC values for the aquatic vector mortality rate ($\mu_{I}$), the aquatic vector maturation rate ($p$ ), the rainfall carrying capacity ($\upsilon$), the optimal rainfall ($m_{o}$) and the rainfall history interval ($t_{d}$). **A**) Shows PRCC values of model parameters with respect to the population dynamics of active adult mosquitoes, while **B**) shows the sensitivity of the parameters with respect to the population dynamics of the aestivating mosquitoes. A positive PRCC value suggests that increasing the corresponding parameter will increase the population density of mosquitoes while a negative PRCC value will achieve the opposite.

**Figure S5: Mosquito seasonal net reproduction number.** The net reproduction numbers change with the rainfall pattern. High reproduction numbers are predicted during peak wet season, while the lowest numbers are predicted in the dry season. Panel **A**, shows the time kinetics of the mosquito daily net reproduction number for the model $M_{0}$/H:0, Panel **B** model $M_{1}$/H:1. Panels **C** and **D** show the effective reproduction numbers for assumptions H:2 and H:3 using model $M_{2}$.

**References**

1. Baber I, Keita M, Sogoba N, Konate M, Diallo M, Doumbia S, et al. Population size and migration of *Anopheles gambiae* in the Bancoumana Region of Mali and their significance for efficient vector control. PLoS One. 2010;5 4:e10270. <https://www.ncbi.nlm.nih.gov/pubmed/20422013>.

2. Adamou A, Dao A, Timbine S, Kassogué Y, Diallo M, Traoré SF, et al. The contribution of aestivating mosquitoes to the persistence of *Anopheles gambiae* in the Sahel. Malar J. 2011;10 1:151.

3. Omer SM, Cloudsley-Thompson J. Survival of female *Anopheles gambiae* Giles through a 9-month dry season in Sudan. Bull World Health Organ. 1970;42 2:319.
